# Assessment and Treatment in Autism Spectrum Disorders: A Focus on Genetics and Psychiatry

**DOI:** 10.1155/2012/242537

**Published:** 2012-05-31

**Authors:** Merlin G. Butler, Erin L. Youngs, Jennifer L. Roberts, Jessica A. Hellings

**Affiliations:** ^1^Department of Psychiatry and Behavioral Sciences, Kansas University Medical Center, 3901 Rainbow Blvd., MS4015, Kansas City, KS 66160, USA; ^2^Department of Pediatrics, Kansas University Medical Center, Kansas City, KS 66160, USA; ^3^Saint Luke's Cancer Institute, Saint Luke's Health System, Kansas City, MO 64111, USA

## Abstract

Autism spectrum disorders (ASDs) are neurobehavioral disorders characterized by abnormalities in three behavioral domains including social interaction, impaired communication, and repetitive stereotypic behaviors. ASD affects approximately 1% of children and is on the rise with significant genetic mechanisms underlying these disorders. We review the current understanding of the role of genetic and metabolic factors contributing to ASD with the use of new genetic technology. Fifty percent is diagnosed with chromosomal abnormalities, small DNA deletions/duplications, single-gene conditions, or metabolic disturbances. Genetic evaluation is discussed along with psychiatric treatment and approaches for selection of medication to treat associated challenging behaviors or comorbidities seen in ASD. We emphasize the importance of prioritizing treatment based on target symptom clusters and in what order for individuals with ASD, as the treatment may vary from patient to patient.

## 1. Introduction

Classical autism which was first described in 1943 [1] belongs to a group of heterogeneous disorders known as autism spectrum disorders (ASD). These neurobehavioral disorders are characterized by abnormalities in three behavioral domains including disturbances in social interaction, impaired communication skills, and repetitive stereotypic behaviors with an onset recognized prior to 3 years of age [2]. ASD includes not only classical autism (autistic disorder) but also asperger disorder (high functioning) and pervasive developmental disorder not otherwise specified (PDD-NOS) [2–6]. The American Academy of Pediatrics recommends autism screening of all infants and toddlers for early identification and intervention by at least 12 months of age and again at 24 months. Several validated rating scales are helpful in establishing the diagnosis, including Autism Diagnostic Interview-Revised (ADI-R) and the Autism Diagnostic Observation Schedule (ADOS), in combination with clinical presentation [7–9]. Specialist assessments and work-ups are available usually at university hospitals and university-affiliated programs and ideally should include regular visits at least annually depending on the chief complaint with a psychologist specializing in ASD, a psychiatrist to examine for treatable symptom presentations such as inattention, a neurologist for seizure assessment and brain imaging to exclude anatomical abnormalities, and a clinical geneticist to identify a known genetic syndrome causing autism, genetic counseling issues, and appropriate genetic testing for family members (now or in the future) at risk for inheriting genetic defects causing autism. Professionals specializing in complementary and alternative treatments are becoming increasingly utilized, although more studies are needed.

Symptoms of ASD usually begin in early childhood and are frequently accompanied by intellectual disability (ID) (75%), dysmorphic features and epilepsy (25%), and occasionally MRI and EEG abnormalities [10, 11]. Microcephaly is reported in about 10% of children with autism [12, 13] and may be associated with a poor prognosis while macrocephaly is reported in 20–40% of autistic children [14, 15]. Mutations of the *PTEN* tumor suppressor gene have been reported in subjects with extreme macrocephaly and autism [16]. Brain imaging shows a larger brain volume particularly in the frontal lobes, while the occipital lobes are smaller in size [17–20]. The etiology of ASD is complex and involves genes and the environment (epigenetics), including the uterine environment and the mitochondria. ASD affects about 1 individual in 100 live births [21] and is on the increase with a higher prevalence than reported for congenital brain malformations or Down syndrome. Better awareness and more accurate genetic and biochemical testing are now available leading to earlier diagnosis and potential treatments at the molecular level. Approximately 30% of individuals with ASD and/or ID also requires psychological and psychiatric treatments, for behavioral problems including hyperactivity, impulsivity, inattention, aggression, property destruction, self-injury, mood disorders, psychosis, and tic disorders [22, 23].

Family studies suggest that genetic factors contribute significantly to autism (up to 90%) [24]. The recurrence risk for ASD varies by gender for the second child to be affected (4% if the first child affected is female and 7% if a male) [25–27]. The recurrence rate increases to 25–30% if the second child is also diagnosed with ASD. Single-gene conditions are identifiable in less than one-fifth of subjects with ASD, while the remaining subjects have other genetic or multigenic causes and/or epigenetic influences. Epigenetics refers to environmental factors such as nutrition, toxins, or infections that alter gene expression without changing the DNA sequence [28–30]. Genome-wide linkage and association studies have identified at least 175 loci in all chromosomes excluding the Y chromosome [31]. Routine cytogenetic studies have identified deletions, duplications, and translocations in individuals with autism, supported by early linkage studies specifically for chromosome regions 2q, 3p, 3q, 7q, 8q, 11p, 15q, 16p, 17q, 19p, Xp, and Xq [31, 32].

Tuberous sclerosis and fragile X syndrome are the most common single gene or monogenic conditions associated with ASD but yet account for less than 10% of all cases (see Table 1) [13, 29, 32]. The most common chromosomal abnormality in nonsyndromal autism is a maternal duplication of the chromosome 15q11-q13 region, accounting for 5% of patients and involves important candidate genes including *UBE3A*, *GABRA5*, and *GABRB3*. Deletions in chromosome 16p11.2 and 22q11 regions account for another 1% of cases [33–36]. X chromosome skewness has also been implicated in autism whereby the X chromosome in affected females shows a nonrandom inactivation pattern (e.g., 80% : 20%) [37]. The incidence of ASD in the past 30 years has increased possibly due to improved identification and better awareness but indicates environmental factors are acting on essentially unchanged genetic predispositions since changes in genetic material are unlikely to occur in such a short period of time. An ongoing and dynamic interplay between the gut, the immune system, and brain development is being elucidated [38].

## 2. Genetic Factors Contributing to Autism

Advances in genetic testing and syndromic recognition of individuals with ASD result in the ability to identify a cause in about 50% of cases. For example, Schaefer et al. in 2006 and later in 2008 [27, 39] used preevaluation assessments and a three-tier clinical genetic approach to identify causes in children diagnosed with ASD and found positive genetic findings in about 40% of cases. These included 5% with a high-resolution chromosomal abnormality, 5% with fragile X syndrome, 5% with Rett syndrome, 3% with *PTEN* gene mutations, approximately 10% with other genetic syndromes (e.g., tuberous sclerosis), and 10% with structural genomic deletions or duplications using early versions of chromosomal microarrays. An additional 10% yield can be obtained with newer microarray technology [40]. Children with ASD are reported with microdeletions and duplications using newer techniques involving chromosome regions 1q24.2, 2q37.3, 3p26.2, 4q34.2, 6q24.3, 7q35, 13q13.2-q22, 15q11-q13, 15q22, 16p11.2, 17p11.2, 22q11, and Xp22 [13]. Additional genetic and cytogenetic conditions and factors associated with ASD are summarized in recent reviews [30, 31, 34, 41, 42].

Newly developed DNA or chromosomal microarrays can identify abnormalities 100 times smaller than seen with high-resolution chromosome analysis. Surveys using these new genetic testing approaches in ASD have been reported including a study performed by Shen et al. [43] on 933 patients with ASD. They reported their experience using standard karyotype analysis, fragile X DNA testing, and chromosomal microarrays and found abnormal karyotypes in 2.2.%, abnormal fragile X testing in 0.5%, and microdeletions or microduplications using chromosomal microarrays in 18.2% of subjects. Most copy number changes were unique except for recurrent deletions or duplications of chromosome 16p11.2 [36] and for chromosome 15q13.2q-13.3 [44]. Furthermore, Wang et al. [45] reported a genome-wide association study on 4300 affected children with ASD and 6500 controls of European ancestry. They found a strong association with six nucleotide polymorphisms located between cadherin 10 (*CDH10*) and cadherin 9 (*CDH9*) genes on chromosome 5 which code for neuronal cell-adhesion molecules. About 175 known or candidate genes have been identified and associated with ASD and represent almost all human chromosomes [31]. These genes include several members of the neuroligin, neurexin, GABA receptor, cadherin, and SHANK gene families. Other genes code for neurotransmitters, their receptors and transporters, oncogenes, brain-derived hormones, signaling and ubiquitin pathway proteins, and neuronal cell-adhesion molecules [31, 46].

Copy number variants (CNVs) or structural genomic changes (microdeletions or microduplications at the DNA level) have been studied with microarrays in the sporadic form (simplex) of autism compared with a positive family history (multiplex) due to single-gene mutations. For example, Sebat et al. [47] examined 165 individuals with autism grouped into 118 simplex and 47 multiplex families compared with controls using chromosomal microarrays with DNA probes and comparative genomic hybridization. They reported that 10% of the individuals with autism from simplex families had CNVs while only 3% of individuals with autism from multiplex families showed CNVs compared with 1% seen in normally developing children studied as controls. The majority of the CNVs were of the deletion type.

## 3. Metabolic Factors Contributing to Autism

Next generation DNA sequencing allows for rapid and efficient detection of mutations at the nuclear and mitochondrial DNA (mtDNA) level in human investigations and is potentially more informative than chromosomal microarray analysis. This technology has advanced to the point where it is available in the clinical setting for individuals with autism presenting with biochemical disturbances involving the mitochondria [48, 49]. The mitochondria are intracellular organelles found in the cytoplasm which play a crucial role in adenosine 5′-triphosphate (ATP) production through oxidative phosphorylation [50, 51]. The latter process is carried out by the electron transport chain made up of complexes I, II, III, and IV situated in the inner membrane of the mitochondria containing about 100 proteins encoded by both nuclear and mitochondrial DNA [52, 53] required to convert food sources to cellular energy. The mitochondrial genome encodes 13 of these 100 proteins [54]. Variations in mitochondrial function can impact on energy levels and influence brain development and activity. Human mitochondrial DNA (mtDNA) is a circular double-stranded DNA molecule contained within the mitochondrion and inherited solely from the mother. Each mitochondrion contains 2–10 mtDNA copies. In humans, 100–10,000 separate copies of mtDNA are usually present per cell [50, 51, 54].

Inborn errors of metabolism may contribute significantly to the causation of ASD with enzyme deficiencies leading to an accumulation of substances that can cause toxic effects on the developing brain. A common example is phenylketonuria leading to excessive phenylalanine levels, intellectual disability, and ASD, if not diet controlled. High lactate levels are also reported in about one in five children with ASD, further supporting the role of the mitochondria in energy metabolism and brain development [55]. Mitochondrial disturbances include a depletion type or reduced number of mitochondria per cell, with a decreased quantity of mtDNA, or mtDNA mutations producing defects in biochemical reactions within the mitochondria and individual cells [30, 56]. A subset of individuals with ASD can manifest copy number variation or have small DNA deletions/duplications which are detectable with mitochondrial genome microarrays. Medical treatments are now available to specifically target the biochemical defect in the mitochondria, if identified, to improve function, bioenergy utilization and lessen the neurological insults that might occur if left untreated.

## 4. Diagnostic Approach for Autism Spectrum Disorders

### 4.1. Initial Clinical Evaluation

A healthcare professional interviews the parent or caregiver regarding presenting problems, history of these problems, a three-generation family history, developmental milestones and abnormal behaviors of the child, a medical and surgical history, and any current treatments. He or she then performs physical and mental status examinations and orders tests as appropriate, including blood tests to check lead levels, thyroid function, lactate, pyruvate and cholesterol levels, and urine for organic acids and makes referrals for imaging, neurological and genetic work-ups. The ADI-R and ADOS are used primarily at academic centers to confirm the ASD diagnoses, although as yet asperger disorder remains a clinical diagnosis. Future diagnostic issues are discussed in a recent editorial [57].

 Applied behavior analysis (ABA) intervention [58] performed for 40 hours per week is considered a validated intervention for children with ASD, funded by some state insurance plans, and is increasingly gaining recognition as an in-home behavioral intervention program. Educational, speech, and occupational therapies are mainstream interventions that should be established for the specific needs of each individual. Evaluation of psychiatric problems may be requested by the school or parents, resulting in further psychiatric and psychological assessments and treatment. Parent training and social skills are also key to achieving improvements in behavior and functioning.

### 4.2. Genetic Work-Up

To increase the diagnostic yield in individuals with ASD presenting for genetic services, Schaefer and Lutz [27] proposed a three-tier approach. This approach is based on a preevaluation screen for confirmation of the diagnosis of autism by review of medical and laboratory records. After this initial screening to identify known syndromes with or without dysmorphic features (e.g., birth marks), a targeted screen is recommended to include viral titers (e.g., rubella), metabolic screening (urine for organic acids and mucopolysaccharides, plasma lactate, and aminoacid levels), and DNA testing for fragile X syndrome in males. The second tier of testing consists of DNA analysis for Rett syndrome in females and males, chromosomal microarrays, and *PTEN* gene mutation screening if head size of the patient is >2 SD. The third tier of screening includes a brain MRI, if not previously done, serum and urine uric acid levels, and assays for adenylate succinase deficiency. With the advent of new testing for mitochondrial function including biochemical genetic studies and chromosomal microarrays, mitochondrial genome screening and function should be undertaken if the above testing protocols are not diagnostic [40]. Therefore, the diagnostic evaluation for autism should include a clinical genetic evaluation with collection of detailed family and medical histories, a genetic consultation to evaluate for clinical genetic syndromes (e.g., dysmorphic features, birth marks, and macrocephaly), cytogenetic problems (chromosomes, FISH), and DNA testing (fragile X, Rett and *PTEN* mutations), chromosomal microarray studies for structural genomic and mtDNA problems, and biochemical (organic, amino-, and fatty acid levels) and mitochondrial function (lactate/pyruvate) assays. The following clinical report presents an individual with ASD who was diagnosed as an adult with a genetic defect accounting for his clinical presentation using new genetic testing technology with chromosomal microarray testing.

**Clinical Case Report: 16p13.2 Duplication and Involvement of the *A2BP1 *Gene Identified with Chromosomal Microarray in an Adult Male with Autism Spectrum Disorder**

We describe an individual with ASD who was found to have a 16p13.2 duplication (53 kb in size) involving the ataxin-2-binding protein-1 (*A2BP1*)  gene,  detected by chromosomal microarray analysis. To our knowledge, he is the first individual reported to have an autism spectrum disorder with this chromosome 16 microduplication. This gene has been reported previously to cause autism but due to a microdeletion.

The proband was diagnosed in his twenties with ASD and intermittent explosive disorder based on DSM IV-TR criteria [2] and responded to risperidone and supportive treatment. The vineland adaptive behavior scale showed an adaptive behavior composite standard score of 80, a communication domain score of 74, daily living skills domain score of 100 and a socialization domain score of 81. He was initially referred to psychiatry after he had assaulted a lower functioning peer who had inadvertently bumped into him in a doorway of the workshop where he was then working. The psychologist who interviewed him at that time sought a psychiatric consultation and suggested he was possibly schizophrenic. His explanation for injuring someone with limited walking abilities was, “America is a free country and I have a right to defend myself.” This behavior appeared paranoid in nature. On psychiatric evaluation, he manifested very good verbal skills, significant perseveration, markedly impaired social insight rather than psychosis, and features of asperger disorder. Another problem identified was disturbing the neighbors by knocking on their door when wanting to know the time, prior to education and support services being made available for him to cope with his needs.

Medically, his history included a right-bundle branch block of his heart, hypertension, acne, and myopia. He had graduated from high school in special education classes and later, briefly attended a technical college. The family history was negative for similarly affected individuals, genetic disorders, or consanguinity. The parents were deceased. Initially, he was treated for laughing spells by a neurologist with antiseizure medications; however, after years of treatment, a negative EEG and normal MRI were reported with no change in status. The antiseizure medication was tapered and was discontinued without problems. Treatment with risperidone at 3.5 mg a day has largely resolved his explosive outbursts. He is monitored for extrapyramidal side effects, tardive dyskinesia, and metabolic syndrome at his quarterly psychiatry clinic visits. Laboratory tests performed every 6 months include a CBC, chemistry profile, fasting lipid panel, and prolactin level.

**Figure 1:**
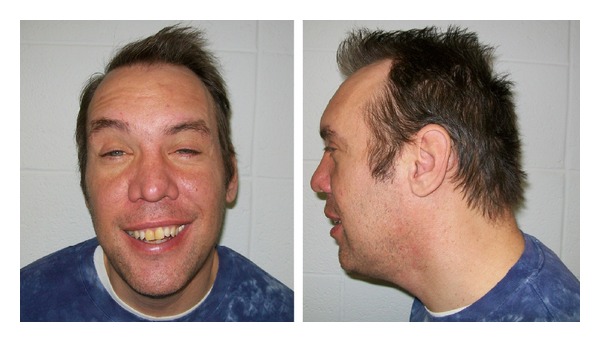
Frontal and profile facial views of the proband at 41 years of age.

On genetic evaluation at 41 years of age, he was nondysmorphic, cooperative with verbal, monotone, pressured speech, and noticeable perseveration. He had mild grandiosity with limited insight and judgement. His height was 176 cm (50th centile), weight was 89.5 kg (90th centile), and head circumference was 58.6 cm (98th centile). Inner canthal distance was 3.2 cm (70th centile), inner pupillary distance was 6.3 cm (97th centile), and outer canthal distance was 8.8 cm (60th centile). Palpebral fissure length was 2.8 cm (75th centile). His ear lobes were attached with an ear length of 7.2 cm (97th centile). Acne was noted on his face, chest and back but otherwise the skin was normal in appearance and without birthmarks. Hand length was 18.9 cm (90th centile) and middle finger length was 8.0 cm (75th centile) but without transverse palmar creases. The remainder of the examination was within normal limits (Figure 1). He is employed part time, lives alone, and receives social services for transportation, house-hold maintenance and activities and for financial arrangements.

**Figure 2:**
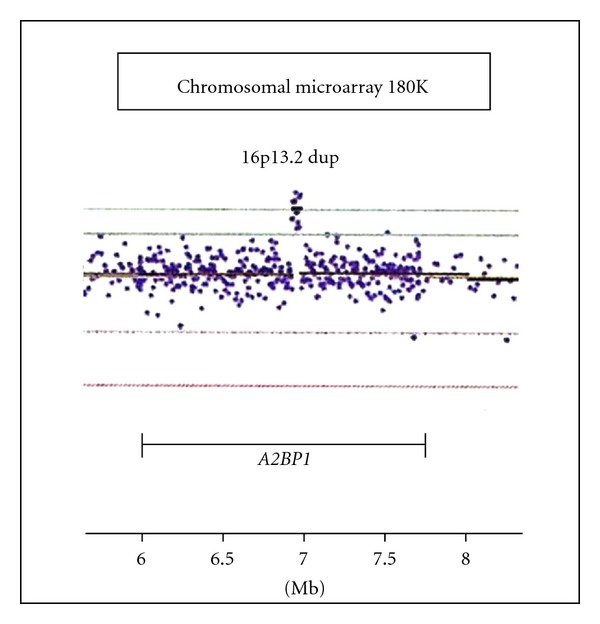
Chromosomal microarray analysis of the proband showed a 53 kb duplication of the 16p13.2 region occurring at 6,936,805 to 6,990,017 bp from the p-terminus which includes a partial duplication of the *A2BP1* gene.

Chromosomal microarray analysis (aCGH) was performed by CMDX Laboratories (Irvine, CA, USA) using the 180 K Oligo HD Scan to rule out possible microdeletions or microduplications of the genome. The aCGH study showed a 16p13.2 duplication at chromosome position 6,936,805–6,990,017 base pairs from the p-terminus involving the middle portion of the *A2BP1 *gene (Figure 2). The *A2BP1 *gene has been reported previously as a candidate gene for autism [31, 59] and disruption (loss) of the gene identified in patients with autism [59, 60]. However, there are no reports, to our knowledge, of individuals with an autism spectrum disorder and a gain or partial duplication of the *A2BP1 *gene. Specifically, Martin et al. [59] reported a girl with autism, global developmental delays, epilepsy, hypotonia, an uneven gait, and mild facial dysmorphism. Cytogenetic and FISH analysis performed in this girl showed an unbalanced *de novo* translocation involving chromosomes 15 and 16 with a 160 kb deletion of chromosome 16 resulting in the loss of exon 1 of the *A2BP1* gene with decreased mRNA expression in the lymphocytes.

Our proband was more mildly affected than those reported previously with a deletion involving the *A2BP1 *gene. This may be explained by our proband having a gain of function rather than a loss of function of the gene, therefore having a milder phenotype. Furthermore, our proband illustrates the importance of using advanced genetic testing (chromosomal microarrays) to identify genetic defects which are beyond the resolution of routine cytogenetic or chromosome studies.

## 5. Psychiatric Treatment of Associated Challenging Behaviors/Comorbidity in ASD

Untreated challenging behaviors are likely to interfere with the individual's development as well as educational inclusion and family life. Many individuals with ASD manifest behavioral problems that can be identified using DSM-IV-TR [2] diagnostic symptom clusters to guide choice of comorbid diagnosis and related treatment selections. Principles of treatment selection include “First do no harm,” “Start low and go slow with any medications,” and “Individualize patient care.” For example, in terms of psychiatric treatment, a low dose of stimulant medication such as methylphenidate [61] or dextroamphetamine [62] may be tried initially for a person with ASD and mild to moderate symptoms of inattention, impulsivity, and/or hyperactivity, while a low dose of atomoxetine [63, 64] may be more suitable if the individual also has significant self-injurious behaviors, since stimulants could worsen anxiety and self-injury.

Individuals presenting with aggression, property destruction, or self-injury as main symptoms are examples that may benefit from first-line treatment with low doses of risperidone or aripiprazole prior to any treatments for hyperactivity, if needed [61, 65–67]. Once a trend towards improvement is obtained with medication, the dose may be cautiously increased to achieve significant improvement, while any side effects are closely monitored. Combination treatments may be necessary in order to minimize side effects and maximize benefits on behavior, although additional studies are needed in this population and in general [68].

Comorbid diagnoses are helpful in guiding the treatment focus, while bearing in mind a likely commonality between neurobiological causes of the ASD symptoms as well as any psychiatric symptom behaviors or clusters. Common symptom clusters in ASD include the hyperactive-inattentive impulsive-distractible cluster, the compulsive-sameness-explosive symptom cluster, tics and tourette syndrome, and a mood disorder symptom cluster which may be depressive or bipolar in nature [22, 69]. It is not uncommon for one or more of these clusters to cooccur, emphasizing the need to prioritize treatment trials based on symptoms to target and in what order.

Hyperactivity across multiple settings, including home and school, may be the most obvious symptom suggesting the hyperactive-inattentive impulsive-distractible symptom cluster [70–72]. One diagnostic difficulty is that hyperactivity in youth without disabilities is known to diminish in comparison with other symptoms of attention deficit hyperactivity disorder (ADHD) in the early teenage years, in which impulsivity and its association with ADHD treatment response may be missed [73]. Impulsivity often presents as hitting, kicking, biting, cussing, running off, and throwing objects but responds to treatments indicated for ADHD, although combination treatments may be needed, for example, a stimulant medication such as dextroamphetamine together with low-dose atomoxetine. Inattention alone is easily missed clinically, but if identified and treated, will likely improve developmental progress. Methylphenidate may be useful, starting at 1 mg/kg/day in 3 divided doses [61, 74]. Dextroamphetamine is longer acting than methylphenidate, more potent and is started at 0.5 mg/kg/day, in morning and midday doses, with a possible 4pm half dose [62].

While long-acting stimulant preparations of these two main stimulants are available, studies in individuals with ASD are lacking. Clinical practice suggests that the side effects of appetite decrease, anxiety, and insomnia may also show greater worsening with long-acting rather than with short-acting stimulants. Atomoxetine is started at 0.5 mg/kg/day and increased gradually as tolerated to approximately 1 mg/kg/day or up to 1.5 mg/kg/day [63, 64], although lower doses may provide meaningful help. Seizures, headaches, behavioral activation, appetite decrease, and cardiovascular side effects require close monitoring. Atomoxetine also has mood elevating properties, and thus may be a useful choice over a selective serotonin uptake inhibitor (SSRI), in the presence of depressive and attentional symptom clusters.

Another useful medication, albeit requiring caution, and close monitoring for the hyperactive-inattention symptom cluster is amitriptyline, but prospective studies are warranted [75, 76]. Our experience suggests that this tricyclic antidepressant, amitriptyline, is more effective in persons with ASD than is clomipramine [77], imipramine, or desipramine, in the treatment of hyperactive, aggressive children. Amitriptyline is safe in low doses with trough blood levels at 100 to 150 mcg/dL, according to a recent chart review of 50 patients [76]. Both clomipramine and desipramine are associated with superior response over placebo in the study reported by Gordon et al. [77] in a 10-week randomized crossover study in seven children with ASD. Quarterly EKG monitoring is necessary to monitor for QTc prolongation, as well as documented warnings to parents to lock medications away related to overdose toxicity.

As noted above, low doses of stimulants and risperidone or aripiprazole may be used in combination treatments, necessitating close monitoring for side effects. Other treatments for this symptom cluster include the use of alpha agonist drugs, clonidine and guanfacine, and long-acting preparations thereof. Mild improvements in hyperactivity and irritability were achieved in a double-blind crossover study of clonidine and placebo in 8 children with ASD [78, 79], although eventual drug tolerance, sedation, and low blood pressure were problematic effects. Posey and McDougle [80] also performed a retrospective review of 80 children with ASD treated with guanfacine, in which 19 of 80 were rated as responders. An open-label, prospective study by Scahill et al. [81] found that 48% of 25 children with ASD and ADHD symptoms were guanfacin responders.

Individuals manifesting the compulsive-sameness-explosive symptom cluster often engage in arranging objects, hoarding, and repetitive behaviors, to an extent that interferes with their own and others' functioning [82]. Tantrums and explosive behaviors are common if the individuals' routines are disrupted or prevented, resulting in screaming, aggression, self-injury, and property destruction if severe. Impulsivity, associated with the hyperactivity symptom cluster or a bipolar mood disorder symptom cluster may require appropriate concomitant medication treatments and may be missed if the compulsive symptoms are severe in nature. Associated bipolar mood disorder symptoms include irritable mood, laughing or crying spells, pacing, sexual preoccupation, and rapid flaring into aggression [69]. Mood stabilizing medications such as antiseizure medications, lithium, and/or antipsychotics may be helpful in the latter cases.

While SSRIs are a mainstay of treatment for individuals with obsessive compulsive disorder in the general population, activation, and lack of response are problematic in SSRI treatments for individuals with ASD [83]. In our experience SSRIs may be helpful mostly in mild cases without other comorbidity. A recent multisite randomized, placebo-controlled trial of citalopram showed no significant improvement over placebo for repetitive behaviors rated on the modified Yale-Brown Obsessive Compulsive scale [84]. Two open-label studies showed benefit of sertraline and fluoxetine in individuals with ASD, although behavioral activation can be limiting [85, 86] and dose related. Fluvoxamine reduced aggression and compulsive behaviors by 50% in a randomized, double-blind placebo controlled study of 30 adults with ASD [87]. In addition, drug interactions require vigilance in SSRI prescription, particularly of paroxetine, fluoxetine, and sertraline due to inhibition of the cytochrome P450 enzyme system which is responsible for breakdown of many psychotropic and nonpsychotropic medications. In addition, sexual side effects of SSRIs may be difficult to elicit in individuals who lack expressive language and may impede sexual functioning and satisfaction, also to be considered in individuals without a sexual partner.

Mood disorder cluster symptoms may be depressive or identified on the bipolar spectrum. Depressive symptoms include sadness and low mood, withdrawn behavior, insomnia or excessive sleeping, appetite increase or decrease, and suicidal thoughts or behavior. Low-dose antidepressant medications and psychotherapy are appropriate treatments. Bipolar mood disorder symptoms include irritability, euphoria, or mixed states with both laughing and crying spells. Associated externalizing behaviors include aggression, property destruction, and elopement. In individuals with ASD, bipolar disorder is more often atypical, chronic, mixed, or rapid cycling [69]. Constant loud vocalizations, pressured speech, insomnia, and aggression together with constant pacing may occur [88]. While atypical antipsychotics are potent mood stabilizers with rapid onset of effects, antiseizure mood stabilizing agents may also be effective and used as a first-line treatment in mild or moderate cases. These include valproic acid (VPA), gabapentin in combination with VPA [89], and carbamazepine. Atypical antipsychotics include risperidone, which is the most studied in this population [65, 66], aripiprazole [90], olanzapine [91], quetiapine [92, 93], and ziprasidone [94, 95]. All of these medications carry a black box label (FDA warning) for weight gain, metabolic syndrome, and type II diabetes. Risperidone may further cause prolactin elevation, more pronounced in females, resulting in enlarged breasts (gynaecomastia), lactation, menstrual irregularities, and possible changes in bone and sexual development [96]. Low doses of an antipsychotic drug in combination with antiseizure medications or lithium may also be beneficial for resistant cases, with close monitoring of metabolic indicators of weight, blood pressure, serum lipids, and HbA1c. In addition, risperidone may be effective in low doses for self-injury [65]. Atypical antipsychotics are also prescribed as first-line treatments for irritability and aggression, although clinicians should carefully examine for missed or undertreated hyperactive-inattentive impulsive-distractible symptoms which are likely to respond more significantly to ADHD medications, as discussed above.

Several other medications have been studied so far targeting the core social relating impairments in ASD [97]. These include memantine [98, 99], amantadine [100], D-cycloserine [101, 102], lamotrigine [103], secretin [104, 105], and naltrexone [106–109]. All of these agents produced negative or mixed results. In addition, an oxytocin spray is now available, but studies are needed to clarify its effects, if any, in individuals with ASD.

In conclusion, we describe our current approach for evaluation and treatment of individuals with developmental delays and associated behavior problems in ASD. Early assessment and work-up, although laborious and requiring multidisciplinary consultation, produce the best chances for improved outcomes in children, adolescents, and adults with ASD.

## 6. Future Directions for Research

Advances made in genetic testing coupled with development of bioinformatics and searchable computer genetic variant data bases, subsequent to completion of the Human Genome Project, have led to significant discoveries and recognition of genetic defects in the causation of ASD. Improvements of chromosome microarray technology with combination of probes for both copy number variants and single nucleotide polymorphisms (SNPs) have not only led to enhanced testing capabilities in identifying segmental deletions and duplications in the genome but also the identification of disease-causing genes and their positions within chromosomal regions.

Next generation DNA sequencing of the exons (referred to as exome sequencing) will allow for new discoveries of disease causing SNPs, gene regulatory sequences, or mutations of protein coding genes for both structural and regulatory proteins. Identifying molecular signatures of novel or disturbed gene or exon expression, disease- specific profiles and patterns (i.e., expression heat maps), and recognition of interconnected gene pathways in autism and other psychiatric or aberrant behavioral disorders in the future by using readily available blood elements (e.g., lymphoblasts) should hold promise for treatments with pharmacological agents by increasing (or decreasing) activity of normal (or abnormal) gene function. The study of noncoding RNAs, which control the amount or quantity of gene expression coding for protein production through micro-RNAs and the quality of protein production by specific isoform development by sno-RNAs, will lead to a new area of research and medical therapies in the future for human diseases including ASD.

A problem with research into ASD is that some studies involved only individuals with autistic disorder while others include the broader spectrum of higher functioning subjects with PDD-NOS and asperger disorder. The authors of the new DSM-5 plan to group individuals affected by autism into one category of ASD with specifiers of mild, moderate, or severe, which may help to overcome this problem.

In addition, greater emphasis is now placed on discovery of biomarkers, the role of the immune system, and molecular causes of ASD, with a view to reversing core features of autism in the future. Although biotechnology companies and the pharmaceutical industry have been slow to enter this area of research, several companies are now focusing on specific disorders with autism as major features such as fragile X and Rett syndromes.

A large National Institutes of Health (NIH) study is underway focusing on autism and regression with an important promise to clarify the vaccine controversy in this area. The study includes proteomic analysis of cerebrospinal fluid, magnetic resonance imaging, sleep EEGs, and the use of novel treatments such as donepezil, riluzole, and minocycline. Another NIH study is focused on children in whom ASD has reportedly remitted [110].

Applying the knowledge from pharmacogenomics and identifying genes and polymorphisms involved in drug metabolism should also benefit patients treated with psychiatric and behavioral problems in the future. In addition, individuals who are either fast or slow metabolizers based on their microsomal genotype patterns and taking psychotropic drugs may respond differently to medications and produce adverse side effects. Similarly, a better understanding of the metabolic differences that occur with age will further impact on drug dosage and selection of specific drugs for treating behavioral problems seen in ASD. The discovery of new classes of drugs and research on existing drugs for new purposes to treat behavioral problems in patients with ASD are under investigation including clinical trials (e.g., in fragile X syndrome) holding promise for improved therapy and a better quality of life. In addition, the discoveries made in brain imaging such as functional MRI or PET scans in identifying regions of the brain that are disturbed in ASD should allow for new treatment discoveries and applications specific for the altered regions identified. More awareness and knowledge about human disorders and genetic and epigenetic discoveries of the causation of ASD should lessen the burden for patient, family, and society. This includes psychiatric and behavioral problems associated with ASD hopefully leading to new and novel treatment modalities with existing drugs and the discovery of new drugs for therapy.

## Figures and Tables

**Table 1:** Partial list of genetic syndromes associated with autism.

Fragile X syndrome (*FMR1* gene)	Apert syndrome
Rett syndrome (*MECP2* gene)	Williams syndrome
Angelman and Prader-Willi syndromes	Joubert syndrome
(15q11-q13 deletions or rearrangements)	Noonan syndrome
Smith-Lemli-Opitz syndrome	Down syndrome
Smith-Magenis syndrome (17p11.2 deletion)	Turner syndrome
Tuberous sclerosis	Neurofibromatosis
PTEN-gene-mutation-associated disorders	Myotonic dystrophy
(Cowden and Bannayan-Riley-Ruvalcaba syndrome with extreme macrocephaly)	Duchenne muscular dystrophy
Shprintzen/velocardiofacial syndrome	Moebius sequence
(22q11 deletion)	Cohen syndrome
Sotos syndrome	Oculoauriculovertebral spectrum
CHARGE syndrome	Untreated or poorly treated phenylketonuria (PKU)
Hypomelanosis of Ito	Adenylate succinase deficiency
De Lange syndrome	
Mitochondrial dysfunction	

Extracted and modified from G.B. Schaefer and N.J. Mendelsohn, “Genetics evaluation for the etiologic diagnosis of autism spectrum disorders,” *Genetics in Medicine*, vol. 10, pp 4–12, 2008.

## References

[1] Kanner L (1943). Autistic psychopathy in childhood. *Nervous Child*.

[2] American Psychiatric Association (2000). *Diagnostic and Statistical Manual of Mental Disorders*.

[3] Lord C, Risi S, Lambrecht L (2000). The Autism Diagnostic Observation Schedule-Generic: a standard measure of social and communication deficits associated with the spectrum of autism. *Journal of Autism and Developmental Disorders*.

[4] Johnson CP, Myers SM, Lipkin PH (2007). Identification and evaluation of children with autism spectrum disorders. *Pediatrics*.

[5] Hughes JR (2009). Update on autism: a review of 1300 reports published in 2008. *Epilepsy and Behavior*.

[6] Polsek D, Jagatic T, Cepanec M, Hof PR, Simic G (2011). Recent developments in neuropathology of Autism Spectrum disorders. *Translational Neuroscience*.

[7] Le Couteur A, Lord C, Ruter M (2003). *Autism Diagnostic Interview-Revised (ADI-R)*.

[8] Lord C, DiLavore PC, Risi S (2003). *Autism Diagnostic Observation Schedule (ADOS)*.

[9] Constantino JN, Davis SA, Todd RD (2003). Validation of a brief quantitative measure of autistic traits: comparison of the social responsiveness scale with the Autism Diagnostic Interview-Revised. *Journal of Autism and Developmental Disorders*.

[10] Rapin I (1995). Autistic regression and disintegrative disorder: how important the role of epilepsy?. *Seminars in Pediatric Neurology*.

[11] Miles JH, Hillman RE (2000). Value of a clinical morphology examination in autism. *American Journal of Medical Genetics*.

[12] Fombonne E, Rogé B, Claverie J, Courty S, Frémolle J (1999). Microcephaly and macrocephaly in autism. *Journal of Autism and Developmental Disorders*.

[13] Miles JH (2011). Autism spectrum disorders-A genetics review. *Genetics in Medicine*.

[14] Lainhart JE, Piven J, Wzorek M (1997). Macrocephaly in children and adults with autism. *Journal of the American Academy of Child and Adolescent Psychiatry*.

[15] Miles JH, Hadden LL, Takahashi TN (2000). Head circumference is an independent clinical finding associated with autism. *American Journal of Medical Genetics*.

[16] Butler MG, Dazouki MJ, Zhou XP (2005). Subset of individuals with autism spectrum disorders and extreme macrocephaly associated with germline PTEN tumour suppressor gene mutations. *Journal of Medical Genetics*.

[17] Carper RA, Courchesne E (2000). Inverse correlation between frontal lobe and cerebellum sizes in children with autism. *Brain*.

[18] Carper RA, Moses P, Tigue ZD, Courchesne E (2002). Cerebral lobes in autism: early hyperplasia and abnormal age effects. *NeuroImage*.

[19] Mueller S, Keeser D, Reiser MF Functional and structural MR imaging in neuropsychiatric disorders, part 2: application in schizophrenia and autism.

[20] Philip RC, Dauvermann MR, Whalley HC (2012). A systematic review and meta-analysis of the fMRI investigation of autism spectrum disorders. *Neuroscience & Biobehavioral Reviews*.

[21] Rice C (2009). Prevalence of autism spectrum disorders—autism and developmental disabilities monitoring network, United States, 2006. *Morbidity and Mortality Weekly Report*.

[22] Hellings JA (2000). Treatment of comorbid disorders in autism: which regimens are effective and for whom?. *MEDSCAPE Mental Health Website*.

[23] Lecavalier L (2006). Behavioral and emotional problems in young people with pervasive developmental disorders: relative prevalence, effects of subject characteristics, and empirical classification. *Journal of Autism and Developmental Disorders*.

[24] Herman GE, Henninger N, Ratliff-Schaub K, Pastore M, Fitzgerald S, McBride KL (2007). Genetic testing in autism: how much is enough?. *Genetics in Medicine*.

[25] Ritvo ER, Jorde LB, Mason-Brothers A (1989). The UCLA-University of Utah epidemiologic survey of autism: recurrence risk estimates and genetic counseling. *American Journal of Psychiatry*.

[26] Bolton P, Macdonald H, Pickles A (1994). A case-control family history study of autism. *Journal of Child Psychology and Psychiatry and Allied Disciplines*.

[27] Schaefer GB, Lutz RE (2006). Diagnostic yield in the clinical genetic evaluation of autism spectrum disorders. *Genetics in Medicine*.

[28] Chakrabarti S, Fombonne E (2001). Pervasive developmental disorders in preschool children. *JAMA*.

[29] Piven J (1997). The biological basis of autism. *Current Opinion in Neurobiology*.

[30] Dhillon S, Hellings JA, Butler MG (2011). Genetics and mitochondrial abnormalities in autism spectrum disorders: a review. *Current Genomics*.

[31] Holt R, Monaco AP (2011). Links between genetics and pathophysiology in the autism spectrum disorders. *EMBO Molecular Medicine*.

[32] Butler MG, Talebizadeh Z (2006). Genetics of autism with emphasis on affected females. *Progress in Medical Genetics Research*.

[33] Schroer RJ, Phelan MC, Michaelis RC (1998). Autism and maternally derived aberrations of chromosome 15q. *American Journal of Medical Genetics*.

[34] Benvenuto A, Moavero R, Alessandrelli R, Manzi B, Curatolo P (2009). Syndromic autism: causes and pathogenetic pathways. *World Journal of Pediatrics*.

[35] Hempel M, Brugués NR, Wagenstaller J (2009). Microdeletion syndrome 16p11.2-p12.2: clinical and molecular characterization. *American Journal of Medical Genetics, Part A*.

[36] Fernandez BA, Roberts W, Chung B (2010). Phenotypic spectrum associated with de novo and inherited deletions and duplications at 16p11.2 in individuals ascertained for diagnosis of autism spectrum disorder. *Journal of Medical Genetics*.

[37] Talebizadeh Z, Bittel DC, Veatch OJ, Kibiryeva N, Butler MG (2005). Brief report: non-random X chromosome inactivation in females with autism. *Journal of Autism and Developmental Disorders*.

[38] Patterson PH (2011). *Infectious Behavior: Brain-Immune Connections in Autism, Schizophrenia and Depression*.

[39] Schaefer GB, Mendelsohn NJ (2008). Clinical genetics evaluation in identifying the etiology of autism spectrum disorders. *Genetics in Medicine*.

[40] Schaefer GB, Starr L, Pickering D, Skar G, Dehaai K, Sanger WG (2010). Array comparative genomic hybridization findings in a cohort referred for an autism evaluation. *Journal of Child Neurology*.

[41] Wall DP, Esteban FJ, DeLuca TF (2009). Comparative analysis of neurological disorders focuses genome-wide search for autism genes. *Genomics*.

[42] Burnside RD, Pasion R, Mikhail FM (2011). Microdeletion/microduplication of proximal 15q11.2 between BP1 and BP2: a susceptibility region for neurological dysfunction including developmental and language delay. *Human Genetics*.

[43] Shen Y, Dies KA, Holm IA (2010). Clinical genetic testing for patients with autism spectrum disorders. *Pediatrics*.

[44] Miller DT, Shen Y, Weiss LA (2009). Microdeletion/duplication at 15q13.2q13.3 among individuals with features of autism and other neuropsychiatric disorders. *Journal of Medical Genetics*.

[45] Wang K, Zhang H, Ma D (2009). Common genetic variants on 5p14.1 associate with autism spectrum disorders. *Nature*.

[46] Glessner JT, Wang K, Cai G (2009). Autism genome-wide copy number variation reveals ubiquitin and neuronal genes. *Nature*.

[47] Sebat J, Lakshmi B, Malhotra D (2007). Strong association of de novo copy number mutations with autism. *Science*.

[48] Manzi B, Loizzo AL, Giana Grazia G, Curatolo P (2008). Autism and metabolic diseases. *Journal of Child Neurology*.

[49] Weissman JR, Kelley RI, Bauman ML (2008). Mitochondrial disease in autism spectrum disorder patients: a cohort analysis. *PLoS One*.

[50] Wallace DC (1986). Mitochondrial genes and disease. *Hospital Practice*.

[51] Wallace DC (1999). Mitochondrial diseases in man and mouse. *Science*.

[52] Schon EA, Manfredi G (2003). Neuronal degeneration and mitochondrial dysfunction. *The Journal of Clinical Investigation*.

[53] DiMauro S, Schon EA (2003). Mitochondrial respiratory-chain diseases. *The New England Journal of Medicine*.

[54] Spelbrink JN (2010). Functional organization of mammalian mitochondrial DNA in nucleoids: history, recent developments, and future challenges. *IUBMB*.

[55] Correia C, Coutinho AM, Diogo L (2006). Brief report: high frequency of biochemical markers for mitochondrial dysfunction in autism: no association with the mitochondrial aspartate/glutamate carrier SLC25A12 gene. *Journal of Autism and Developmental Disorders*.

[56] Pons R, Andreu AL, Checcarelli N (2004). Mitochondrial DNA abnormalities and autistic spectrum disorders. *Journal of Pediatrics*.

[57] Tanguay PE (2011). Autism in DSM-5. *American Journal of Psychiatry*.

[58] Lovaas OI (2003). *Teaching Individuals with Developmental Delays: Basic Intervention Techniques*.

[59] Martin CL, Duvall JA, Ilkin Y (2007). Cytogenetic and molecular characterization of *A2BP1/FOX1* as a candidate gene for autism. *American Journal of Medical Genetics, Part B*.

[60] Bhalla K, Phillips HA, Crawford J (2004). The de novo chromosome 16 translocations of two patients with abnormal phenotypes (mental retardation and epilepsy) disrupt the *A2BP1* gene. *Journal of Human Genetics*.

[61] Research Units in Pediatric Psychopharmacology (RUPP) (2005). Autism Network: randomized, controlled crossover trial of methylphenidate in pervasive development disorder with hyperactivity. *Archives in General Psychiatry*.

[62] Hellings JA, Tanjim S, Saranga V, Thome A Comorbidity and combination treatments with dextroamphetamine in youth with autism spectrum disorders (ASD) and attention deficit hyperactivity disorder (ADHD).

[63] Posey DJ, Wiegand RE, Wilkerson J, Maynard M, Stigler KA, McDougle CJ (2006). Open-label atomoxetine for attention-deficit/hyperactivity disorder symptoms associated with high-functioning pervasive developmental disorders. *Journal of Child and Adolescent Psychopharmacology*.

[64] Arnold LE, Aman MG, Cook AM (2006). Atomoxetine for hyperactivity in autism spectrum disorders: placebo-controlled crossover pilot trial. *Journal of the American Academy of Child and Adolescent Psychiatry*.

[65] Hellings JA, Zarcone JR, Reese RM (2006). A crossover study of risperidone in children, adolescents and adults with mental retardation. *Journal of Autism and Developmental Disorders*.

[66] Research Units in Pediatric Psychopharmacology (RUPP) (2002). Autism network: risperidone in children with autism and serious behavioral problems. *NEJM*.

[67] Hellings JA, Boehm D, Butler MG, Yeh H, Schroeder SR (2011). Long-term clinical aripiprazole efficacy and weight changes in youth with developmental disabilities including autism spectrum disorders. *Journal of Mental Health Research in Intellectual Disability*.

[68] Tamminga CA (2011). When is polypharmacy an advantage?. *American Journal of Psychiatry*.

[69] Hellings JA (1999). Psychopharmacology of mood disorders in persons with mental retardation and autism. *Mental Retardation and Developmental Disabilities Research Reviews*.

[70] Gadow KD, Sverd J (2006). Attention deficit hyperactivity disorder, chronic tic disorder, and methylphenidate. *Advances in Neurology*.

[71] Goldstein S, Schwebach AJ (2004). The comorbidity of pervasive developmental disorder and attention deficit hyperactivity disorder: results of a retrospective chart review. *Journal of Autism and Developmental Disorders*.

[72] Reirsen AM, Amaral DG, Dawson G, Geschwind DH (2011). Attention-deficit/hyperactivity disorder (ADHD). *Autism Spectrum Disorders*.

[73] Barkley RA, Murphy KR, Fischer M (2007). *ADHD in Adults: What the Science Says*.

[74] Handen BL, Johnson CR, Lubetsky M (2000). Efficacy of methylphenidate among children with autism and symptoms of attention-deficit hyperactivity disorder. *Journal of Autism and Developmental Disorders*.

[75] Lockhart P, Guthrie B (2011). Trends in primary care antidepressant prescribing 1995–2007: a longitudinal population database analysis. *British Journal of General Practice*.

[76] Hellings JA, Thome A, Bhatti I, Smith P, Cook-Wiens G Clinical practice informing drug development targeting ASD.

[77] Gordon CT, State RC, Nelson JE, Hamburger SD, Rapoport JL (1993). A double-blind comparison of clomipramine, desipramine, and placebo in the treatment of autistic disorder. *Archives of General Psychiatry*.

[78] Fankhauser MP, Karumanchi VC, German ML, Yates A, Karumanchi SD (1992). A double-blind, placebo-controlled study of the efficacy of transdermal clonidine in autism. *Journal of Clinical Psychiatry*.

[79] Jaselkis CA, Cook EH, Fletcher KE, Leventhal BL (1992). Clonidine treatment of hyperactive and impulsive children with autistic disorder. *Journal of Clinical Psychopharmacology*.

[80] Posey DJ, McDougle CJ (2007). Guanfacine and guanfacine extended release: treatment for ADHD and related disorders. *CNS Drug Reviews*.

[81] Scahill L, Aman MG, McDougle CJ (2006). A prospective open trial of guanfacine in children with pervasive developmental disorders. *Journal of Child and Adolescent Psychopharmacology*.

[82] Taylor BP, Hollander E, Amaral DG, Dawson G, Geschwind DH (2011). Comorbid obsessive-compulsive disorders. *Autism Spectrum Disorders*.

[83] Williams K, Wheeler DM, Silove N, Hazell P (2011). Cochrane review: selective serotonin reuptake inhibitors (SSRIs) for autism spectrum disorders (ASD). *Evidence-Based Child Health*.

[84] King BH, Hollander E, Sikich L (2009). Lack of efficacy of citalopram in children with autism spectrum disorders and high levels of repetitive behavior: citalopram ineffective in children with autism. *Archives of General Psychiatry*.

[85] Cook EH, Rowlett R, Jaselskis C, Leventhal BL (1992). Fluoxetine treatment of children and adults with autistic disorder and mental retardation. *Journal of the American Academy of Child and Adolescent Psychiatry*.

[86] Hellings JA, Kelley LA, Gabrielli WF, Kilgore E, Shah P (1996). Sertraline response in adults with mental retardation and autistic disorder. *Journal of Clinical Psychiatry*.

[87] McDougle CJ, Naylor ST, Cohen DJ, Volkmar FR, Heninger GR, Price LH (1996). A double-blind, placebo-controlled study of fluvoxamine in adults with autistic disorder. *Archives of General Psychiatry*.

[88] Lowry MA (1997). Unmasking mood disorders: recognizing and measuring symptomatic behaviors. *The Habilitative Mental Healthcare Newsletter*.

[89] Hellings JA (2006). Much improved outcome with gabapentin-divalproex combination in adults with bipolar disorders and developmental disabilities. *Journal of Clinical Psychopharmacology*.

[90] Marcus RN, Owen R, Kamen L (2009). A placebo-controlled, fixed-dose study of aripiprazole in children and adolescents with irritability associated with autistic disorder. *Journal of the American Academy of Child and Adolescent Psychiatry*.

[91] Hollander E, Wasserman S, Swanson EN (2006). A double-blind placebo-controlled pilot study of olanzapine in childhood/adolescent pervasive developmental disorder. *Journal of Child and Adolescent Psychopharmacology*.

[92] Findling RL, McNamara NK, Gracious BL (2004). Quetiapine in nine youths with autistic disorder. *Journal of Child and Adolescent Psychopharmacology*.

[93] Hardan AY, Jou RJ, Handen BL (2005). Retrospective study of quetiapine in children and adolescents with pervasive developmental disorders. *Journal of Autism and Developmental Disorders*.

[94] Malone RP, Delaney MA, Hyman SB, Cater JR (2007). Ziprasidone in adolescents with autism: an open-label pilot study. *Journal of Child and Adolescent Psychopharmacology*.

[95] McDougle CJ, Kem DL, Posey DJ (2002). Case series: use of ziprasidone for maladaptive symptoms in youths with autism. *Journal of the American Academy of Child and Adolescent Psychiatry*.

[96] Hellings JA, Zarcone JR, Valdovinos MG, Reese RM, Gaughan E, Schroeder SR (2005). Risperidone-induced prolactin elevation in a prospective study of children, adolescents, and adults with mental retardation and pervasive developmental disorders. *Journal of Child and Adolescent Psychopharmacology*.

[97] Blankenship K, Erickson CA, Stigler KA, Posey DJ, McDougle CJ, Amaral DG, Dawson G, Geschwind DH (2011). Psychopharmacological treatment of autism. *Autism Spectrum Disorders*.

[98] Owley T, Salt J, Guter S (2006). A prospective, open-label trial of memantine in the treatment of cognitive, behavioral, and memory dysfunction in pervasive developmental disorders. *Journal of Child and Adolescent Psychopharmacology*.

[99] Erickson CA, Posey DJ, Stigler KA, Mullett J, Katschke AR, McDougle CJ (2007). A retrospective study of memantine in children and adolescents with pervasive developmental disorders. *Psychopharmacology*.

[100] King BH, Wright DM, Handen BL (2001). Double-blind, placebo-controlled study of amantadine hydrochloride in the treatment of children with autistic disorder. *Journal of the American Academy of Child and Adolescent Psychiatry*.

[101] Goff DC, Tsai G, Levitt J (1999). A placebo-controlled trial of D-cycloserine added to conventional neuroleptics in patients with schizophrenia. *Archives of General Psychiatry*.

[102] Posey DJ, Kem DL, Swiezy NB, Sweeten TL, Wiegand RE, McDougle CJ (2004). A pilot study of D-cycloserine in subjects with autistic disorder. *American Journal of Psychiatry*.

[103] Belsito KM, Law PA, Kirk KS, Landa RJ, Zimmerman AW (2001). Lamotrigine therapy for autistic disorder: a randomized, double-blind, placebo-controlled trial. *Journal of Autism and Developmental Disorders*.

[104] Horvath K, Stefanatos G, Sokolski KN, Wachtel R, Nabors L, Tildon JT (1998). Improved social and language skills after secretin administration in patients with autistic spectrum disorders. *Journal of the Association for Academic Minority Physicians*.

[105] Sturmey P (2005). Secretin is an ineffective treatment for pervasive developmental disabilities: a review of 15 double-blind randomized controlled trials. *Research in Developmental Disabilities*.

[106] Campbell M, Anderson LT, Small AM, Locascio JJ, Lynch NS, Choroco MC (1990). Naltexone in autistic children: a double-blind and placebo-controlled study. *Psychopharmacology Bulletin*.

[107] Campbell M, Anderson LT, Small AM, Adams P, Gonzalez NM, Ernst M (1993). Naltrexone in autistic children: behavioral symptoms and attentional learning. *Journal of the American Academy of Child and Adolescent Psychiatry*.

[108] Willemsen-Swinkels SH, Buitelaar JK, Weijnen FG, van Engeland H (1995). Placebo-controlled acute dosage naltrexone study in young autistic children. *Psychiatry Research*.

[109] Feldman HM, Kolmen BK, Gonzaga AM (1999). Naltrexone and communication skills in young children with autism. *Journal of the American Academy of Child and Adolescent Psychiatry*.

[110] Insel TR, Daniels SA (2011). Future directions: setting priorities to guide the federal research effort. *Autism Spectrum Disorders*.

